# Analysis of cytomegalovirus-specific T-cell responses in patients with hypertension: comparison of assay methods and antigens

**DOI:** 10.1186/s40885-018-0090-8

**Published:** 2018-03-20

**Authors:** Jong-Chan Youn, Jun Yong Kim, Min Kyung Jung, Hee Tae Yu, Su-Hyung Park, In-Cheol Kim, Sun Ki Lee, Suk-Won Choi, Seongwoo Han, Kyu-Hyung Ryu, Sungha Park, Eui-Cheol Shin

**Affiliations:** 1 Division of Cardiology, Dongtan Sacred Heart Hospital, Hallym University College of Medicine, Keunjaebong-gil 7, Hwaseong-si, Gyeonggi-do 18450 Republic of Korea; 2 Laboratory of Immunology and Infectious Diseases, Graduate School of Medical Science and Engineering, KAIST, 291 Daehak-ro, Yuseong-gu, Daejeon, 34141 Republic of Korea; 3 Division of Cardiology, Severance Cardiovascular Hospital, Yonsei University College of Medicine, Seoul, Republic of Korea; 4 Laboratory of Translational Immunology and Vaccinology, Graduate School of Medical Science and Engineering, KAIST, Daejeon, Republic of Korea; 5 Division of Cardiology, Keimyung University Dongsan Medical Center, Daegu, Republic of Korea

**Keywords:** Cytomegalovirus, T cell, Arterial stiffness, Enzyme-linked immunospot (ELISPOT) assay, Intracellular cytokine staining (ICS)

## Abstract

**Background:**

Recent studies suggest an association between cytomegalovirus (CMV) infection and hypertension. In the present study, we used a variety of antigens and different assay methods to investigate the relationship between CMV-specific T-cell responses and arterial stiffness in patients with hypertension.

**Methods:**

To evaluate arterial stiffness, pulse wave velocity (PWV) was measured in 207 hypertensive patients (average age, 63 ± 8 years). To measure CMV pp65 and IE-1-specific T-cell responses, we performed intracellular cytokine staining (ICS) and enzyme-linked immunospot (ELISPOT) assays. We also analyzed CMV-specific T-cell responses against 10 different CMV antigens using ELISPOT assays.

**Results:**

In patients with hypertension, senescent CD8^+^ T-cell frequencies were significantly correlated with arterial stiffness. Moreover, arterial stiffness was independently associated with CMV pp65-specific CD8^+^ T-cell responses as measured by ICS. CMV-specific CD8^+^ T-cell responses measured by ICS and ELISPOT assays showed good agreement and significant correlation with each other. ELISPOT analyses against 10 different CMV antigens revealed a consistent response pattern irrespective of age, gender, and diabetes

**Conclusions:**

CMV pp65-specific CD8^+^ T-cell responses were independently correlated with arterial stiffness in patients with hypertension. Additionally, the results of ICS and ELISPOT assays showed a significant correlation and good agreement with each other. These findings are important for guiding choices regarding the broad clinical application of CMV-specific T-cell response assays in this patient population.

## Background

Recent evidence suggests that infection with cytomegalovirus (CMV) [1–3] and T cell-driven inflammation [4–6] play pathogenic roles in the development of hypertension. Among patients with hypertension, it appears that CMV infection is linked to T cell-driven inflammation via an emerging concept termed immune aging or immunosenescence, in which the functions of immune cells or immune organs are altered with increasing age [7, 8]. CMV infection, one of the most common infections in humans [9], is associated with T-cell senescence [10–12]. CMV antigens are the major antigens responsible for repetitive T-cell stimulation, and the recurrent clonal expansion of CMV-specific T cells induces senescent features in the T-cell population [13–16].

We recently demonstrated that CMV-specific T-cell responses are independently associated with arterial stiffness, suggesting related pathophysiological mechanisms [17]. Thus, investigating CMV-specific T-cell responses will likely help elucidate the immunologic mechanism of hypertension. Here we used a variety of antigens and different assay methods to examine examined the relationship between CMV-specific T-cell responses and arterial stiffness in patients with hypertension.

## Methods

### Study population

The study population comprised 207 hypertensive patients who were registered in the Yonsei Cardiovascular Genome Center cohort. The average age of the enrolled patients was 63.1 ± 8.1 years (range, 37 to 82 years). To assess arterial stiffness, pulse wave velocity (PWV) was measured in all participants. At the time of sampling, all patients were taking anti-hypertensive medication. Hypertension was defined as either a documented systolic blood pressure (SBP) above 140 mmHg, or diastolic blood pressure (DBP) above 90 mmHg, for three visits prior to starting anti-hypertensive medication. Among the participants, 84 (40.6%) had diabetes mellitus (DM), defined as having fasting plasma glucose levels above 126 mg/dL, HbA1c of over 6.5%, or a history of DM treatment. At the time of enrollment, patients underwent a complete physical examination and laboratory assessment. Patients were excluded if they had any of the following conditions: significant systemic disease, debilitating malignant disease, severe hypertension (> 200/140 mmHg), a history of overt chronic inflammatory disease, or use of anti-inflammatory medications. Table 1 summarizes the participants’ baseline clinical characteristics and laboratory data.

**Table 1:** Clinical characteristics and laboratory findings of study subjects

Variables	Values
Age, years	63.1 ± 8.1
Male, n (%)	146 (70.5)
Diabetes, n (%)	84 (40.6)
Hyperlipidemia, n (%)	99 (47.8)
BMI, kg/m^2^	25.6 ± 3.2
SBP, mmHg	133.4 ± 17.0
DBP, mmHg	80.8 ± 10.9
WBC, 10^3^/μL	6.0 ± 1.4
Hemoglobin, mg/dL	14.2 ± 1.4
BUN, mg/dL	17.5 ± 7.0
Creatinine, mg/dL	1.1 ± 0.9
Total cholesterol, mg/dL	154.0 ± 34.6
Triglyceride, mg/dL	129.9 ± 91.3
HDL cholesterol, mg/dL	47.3 ± 10.9
LDL cholesterol, mg/dL	80.8 ± 28.8
FBS, mg/dL	106.7 ± 26.6
hsCRP, mg/dL	1.7 ± 3.3
Uric acid, mg/dL	5.1 ± 1.3
hfPWV, cm/s	1082.5 ± 226.7
baPWV, cm/s	1533.0 ± 304.0
cfPWV, cm/s	925.8 ± 197.6

Data are presented as the mean ± SD or n (%)

*BMI* body mass index, *SBP* systolic blood pressure, *DBP* diastolic blood pressure, *WBC* white blood cell, *BUN* blood urea nitrogen, *HDL* high-density lipoprotein, *LDL* low-density lipoprotein, *FBS*, fasting blood sugar, *hsCRP* high-sensitivity C-reactive protein, *hfPWV* heart-femoral pulse wave velocity, *baPWV* brachial-ankle pulse wave velocity, *cfPWV* carotid-femoral pulse wave velocity

All participants provided informed consent prior to enrollment. This study received prior approval from the Institutional Review Board of the Severance Hospital, Yonsei University College of Medicine, and the study protocol was in accordance with institutional guidelines.

### Pulse wave velocity measurements

PWV was measured using a VP-2000 pulse wave unit (Nippon Colin Ltd., Komaki City, Japan) as previously described [18]. Briefly, with the patients in a supine position, carotid and femoral artery pressure waveforms were recorded using multi-element tonometry sensors positioned at the left carotid and left femoral arteries. Electrodes were placed on both wrists for electrocardiogram monitoring. To detect heart sounds S1 and S2, a microphone was positioned on the left edge of the sternum at the third intercostal space. The waveform analyzer measured the time intervals between S2 and the notch of the carotid pulse wave (Thc), and between the carotid and femoral artery pulse waves (Tcf). Thc and Tcf were summed to determine the time required for a pulse wave to travel from the heart to the femoral artery (Thf). We calculated the distance from the heart to the femoral artery (Lhf), the distance between the heart and ankle (D1), and the distance between the heart and brachium (D2) based on patient height with division by the time interval for the waveform from each measuring point. Using this information, we calculated hfPWV (a marker of central aortic stiffness) as Lhf/Thf, and baPWV (a marker for both central and peripheral arterial stiffness) from the eq. (D1 − D2)/T, where T is the transit time between the right brachial artery wave and right tibial artery wave.

### Immunophenotyping and intracellular cytokine staining (ICS)

Peripheral blood mononuclear cells (PBMCs) were isolated from anti-coagulated blood using Ficoll-Hypaque (GE Healthcare, Uppsala, Sweden) density gradient centrifugation. For surface staining, these PBMCs were incubated for 20 min at 4 °C with the following fluorochrome-conjugated monoclonal antibodies: anti-CD3 (horizon V500), anti-CD4 (PE-Cy7), anti-CD8 (APC-H7), anti-CD28 (APC) (all from BD Biosciences, San Jose, CA, USA), and anti-CD57 (eFluor 450) (Biolegend, USA). To analyze the T cells’ specific antigen reactivity, PBMCs were stimulated for 6 h with overlapping peptide pools covering CMV pp65 or IE-1 (0.6 nmol of each peptide/mL; Miltenyi Biotec). After the first hour of incubation, brefeldin A (GolgiPlug; BD Biosciences) and monensin (GolgiStop; BD Biosciences) were added to induce intracellular cytokine protein accumulation. After the 6-h incubation was complete, the cells were subjected to surface staining with anti-CD3 (horizon V500), anti-CD4 (PerCP-Cy5.5), anti-CD8 (APC-H7), anti-CD28 (horizon V450), and anti-CD57 (APC) antibodies. Then the cells were fixed and permeabilized using a Fixation/Permeabilization Buffer Kit (BD Biosciences), and additionally stained for intracellular cytokines using anti-interferon-γ (FITC-IFN-γ; BD Biosciences). Finally, flow cytometry was performed using an LSR II Flow Cytometer (BD Biosciences), and data were analyzed using FlowJo software (Treestar, San Carlos, CA, USA). The frequencies of antigen-specific cells are presented as the percentage of cells among the total CD8^+^ T-cell population.

### Enzyme-linked immunospot (ELISPOT) assay

PBMCs from 52 patients (33 male; average age, 63 ± 8 years) were available for ELISPOT assays. From these PBMCs, CD8^+^ T cells were positively isolated using microbeads (Miltenyi biotec), and then the CD8^+^ T cell-depleted PBMCs were γ-irradiated with 3000 cGy. Next, the positively isolated CD8^+^ T cells and the irradiated CD8^+^ T cell-depleted PBMCs were mixed at a 1:2 ratio and added to an ELISPOT plate (7.5 × 10^4^ CD8^+^ T cells/100 μl/well). T cells were stimulated using overlapping peptide pools covering ten different CMV antigens: pp65, immediate early-1 (IE-1), immediate early 2 (IE-2), unique long 94 (UL94), pp150, pp71, glycoprotein B (gB), unique short 3 (US3), unique long 48A (UL48A), and unique long 48B (UL48B) (1 μg/mL; all from JPT Peptide Technologies). Positive controls were stimulated with PMA/ionomycin. The ELISPOT plate was incubated for 24 h, and then washed. For spot visualization, we performed a 1-h incubation with biotinylated IFN-γ antibodies (Thermo Fisher Scientific), followed by a 1-h incubation with streptavidine-alkaline phosphatase and an 8-min incubation with color development reagent (BIO-RAD). The spots were counted using an AID ELISPOT reader (Autoimmune Diagnostika GmbH, Germany) with manual modification. The antigen-specific spot number was calculated by subtracting the number of spots in negative control wells from the average number of spots in antigen-stimulated wells. We calculated the frequency of CMV-specific CD8^+^ T cells, and presented this value as the percentage of cells among the total CD8^+^ T-cell population.

### Statistical analysis

Continuous variables are summarized as mean ± standard deviation (SD), and categorical variables as a percentage of the group total. We compared continuous variables using Student’s *t*-test, and paired variables using a paired t-test. Pearson’s correlation analysis was performed to analyze simple correlations between continuous variables. To examine the association of CMV-specific T-cell responses with arterial stiffness, we used multiple linear regression models with hfPWV, cfPWV, faPWV, and baPWV as the dependent variables, and the frequencies of CMV-specific T cells as the main independent variables of interest. Skewed data were modified by using the log if this produced a normal distribution. All *p* values were two-sided and considered significant at the 0.05 level. All statistical analyses were performed using SPSS 18.0. (SPSS Inc., Chicago, IL, USA).

## Results

### Frequencies of CD8^+^CD57^+^ T cells and CD8^+^CD28^null^ T cells were significantly correlated with arterial stiffness in patients with hypertension

**Fig. 1:**
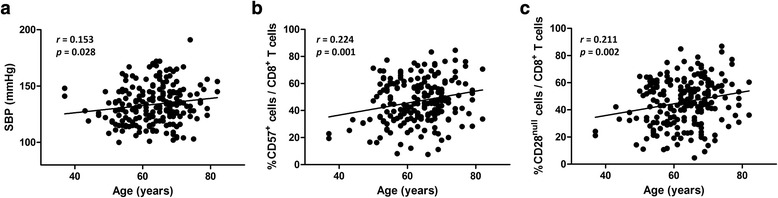
Aging was positively correlated with SBP and senescent CD8^+^ T-cell frequencies. We performed immunophenotyping of PBMCs obtained from 207 hypertensive patients. Increasing age was significantly associated with increased SBP (**a**), and increased frequencies of CD57^+^ cells (**b**) and CD28^null^ cells (**c**) among total CD8^+^ T cells

We investigated how senescent CD8^+^ T-cell frequencies were related to arterial stiffness according to PWV in 207 hypertensive patients. First, we analyzed the age-related changes in SBP and in the frequencies of CD57^+^ and CD28^null^ cells among CD8^+^ T cells. Increasing age was significantly associated with increases in SBP (*p* = 0.028) and in senescent CD8^+^ T-cell frequencies (CD57^+^cell frequency, *p* = 0.001; CD28^null^ cell frequency, *p* = 0.002) (Fig. 1).

**Fig. 2:**
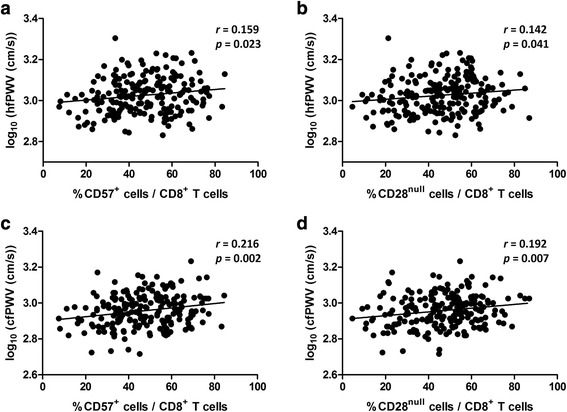
The frequencies of CD57^+^ and CD28^null^ cells among the total CD8^+^ T-cell population were significantly correlated with arterial stiffness as measured by hfPWV (**a**, **b**) and cfPWV (**c**, **d**)

We next examined whether arterial stiffness was correlated with CD57 expression or CD28 loss. The frequencies of CD57^+^ and CD28^null^ T cells among the total CD8^+^ T-cell population were significantly positively correlated with hfPWV (CD57^+^ cells, *p* = 0.023; CD28^null^ cells, *p* = 0.041) and with cfPWV (CD57^+^ cells, *p* = 0.002; CD28^null^ cells, *p* = 0.007) (Fig. 2). Notably, the correlations of cfPWV with the CD57^+^ and CD28^null^ T-cell frequencies remained significant after adjustment for age, gender, DM, SBP, BMI, creatinine, HDL-cholesterol, and hsCRP (CD57^+^ cells, *β* = 0.001, *p* = 0.013; CD28^null^ cells, *β* = 0.001, *p* = 0.020). On the other hand, multivariate analysis revealed that baPWV and faPWV were not significantly correlated with the frequencies of CD8^+^CD57^+^ or CD8^+^CD28^null^ T cells.

### CMV pp65-specific T cells were more frequently detected among senescent CD8^+^ T cells than non-senescent CD8^+^ T cells

**Fig. 3:**
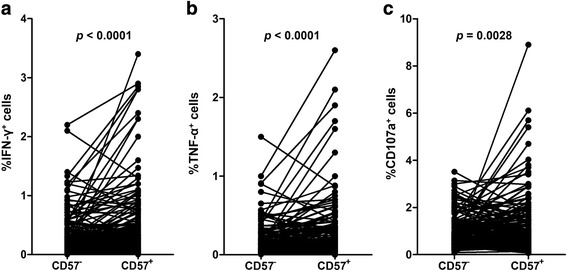
The predominance of CMV pp65-specific CD8^+^ T-cell responses in the CD8^+^CD57^+^ T-cell population. The frequencies of CMV pp65-specific IFN-γ-positive cells (**a**), TNF-α-positive cells (**b**), and CD107a-positive cells (**c**) were compared between the CD8^+^CD57^+^ and CD8^+^CD57^−^ T-cell populations. CMV pp65-specific cells were more frequent among CD8^+^CD57^+^ T cells than among CD8^+^CD57^−^ T cells. The *p* values were calculated using the paired *t*-test.

Since a substantial portion of senescent CD8^+^ T cells are reactive to CMV, we analyzed CMV-specific immune responses in PBMCs from patients with hypertension. To investigate CMV-specific T-cell responses, we stimulated PBMCs with overlapping peptides covering the CMV pp65 antigen. We evaluated T-cell function using intracellular cytokine staining for IFN-γ and TNF-α, and simultaneously evaluated the cytotoxic function of T cells by CD107a staining, which indicates cytotoxic degranulation activity. As previously demonstrated, CMV pp65-specific T cells were more frequent among CD8^+^CD57^+^ T cells than among CD8^+^CD57^−^ T cells (Fig. 3).

### Arterial stiffness was independently correlated with CMV pp65-specific CD8^+^ T-cell response as measured by ICS

**Fig. 4:**
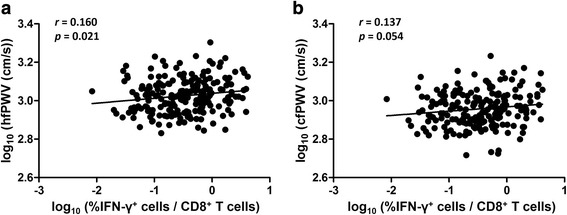
The frequency of CMV pp65-specific IFN-γ^+^CD8^+^ T cells was significantly correlated with arterial stiffness as measured by hfPWV (**a**), but was not significantly correlated with cfPWV (**b**)

We next examined whether arterial stiffness was correlated with CMV pp65-specific CD8^+^ T-cell responses as measured by ICS. Our data showed a significant correlation between hfPWV and the frequency of CMV pp65-specific IFN-γ^+^-CD8^+^ T-cell responses (*p* = 0.021) (Fig. 4). This correlation remained significant after adjustment for age, gender, DM, SBP, BMI, creatinine, HDL cholesterol, and hsCRP (*β* = 0.020, *p* = 0.040). On the other hand, cfPWV was not significantly correlated with CMV pp65-specific IFN-γ^+^CD8^+^ T-cell responses (*p* = 0.054).

### CMV-specific IFN-γ-producing CD8^+^ T-cell responses measured by intracellular cytokine staining and ELISPOT assays showed a significant correlation and good agreement with each other

**Fig. 5:**
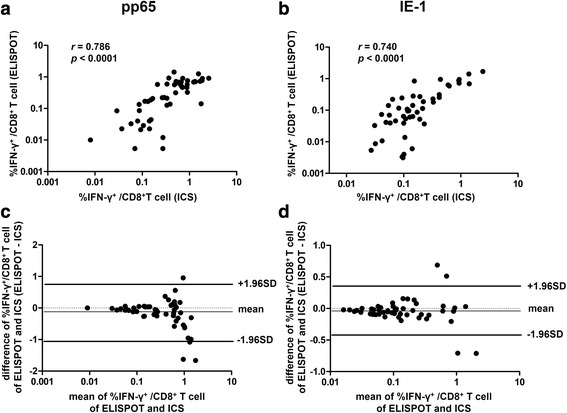
CMV-specific IFN-γ-producing CD8^+^ T-cell responses as measured by ICS and ELISPOT assays showed significant correlation (**a**, **b**) and good agreement (**c**, **d**) with each other

We compared the CMV-specific IFN-γ-producing CD8^+^ T-cell responses in samples from 52 hypertensive patients as measured by ICS versus ELISPOT assays. Simple linear regression analyses revealed that the ICS results were significantly correlated with the ELISPOT assay results, irrespective of CMV antigens (pp65, *p* < 0.0001; IE-1, *p* < 0.0001) (Fig. 5a, b). Bland-Altman analysis further confirmed the good agreement of CMV-specific T-cell responses as determined by ICS and ELISPOT assays (Fig. 5c, d). We also used a paired *t*-test to compare the frequencies of CMV-specific CD8^+^ T cells as measured by ICS and ELISPOT assays, and found no significant difference between the results of the two methods, irrespective of CMV antigens (pp65, *p* = 0.001; IE-1, *p* = 0.003).

### ELISPOT assays for 10 different CMV antigens revealed a consistent response pattern irrespective of age, gender, and diabetes

**Fig. 6:**
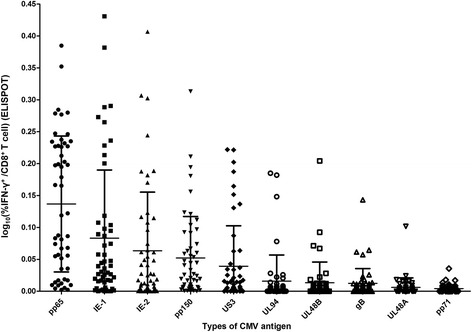
Frequencies of 10 different CMV antigen-specific IFN-γ-producing CD8^+^ T cells among total CD8^+^ T cells from patients with hypertension. From right to left, the results are listed in decreasing order of the mean frequency of each antigen-specific IFN-γ-producing CD8^+^ T-cell group. Bars indicate mean ± SD

**Fig. 7:**
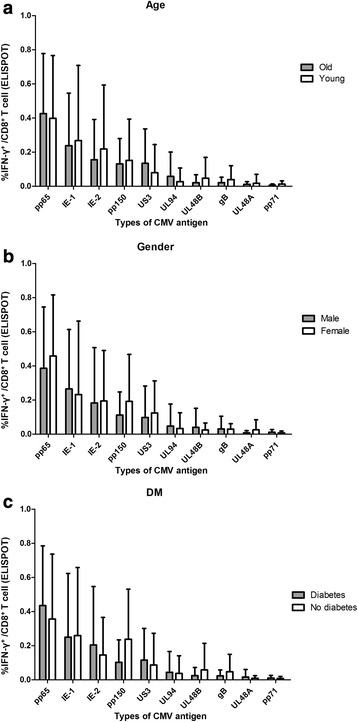
ELISPOT assay for 10 different CMV antigens showed a consistent response pattern, with no significant differences for any antigen types when stratified according to age (**a**), gender (**b**), and diabetes (**c**)

Finally, we investigated CMV-specific IFN-γ-producing CD8^+^ T-cell responses by performing ELISPOT assays using 10 different CMV antigens (pp65, IE-1, IE-2, pp150, US3, UL94, UL48B, gB, UL48A, and pp71). Figure 6 shows the frequencies of IFN-γ^+^ cells among total CD8^+^ T cells from 52 hypertensive patients, with the CMV antigens listed from highest to lowest mean. Among the 10 antigens, CMV pp65 stimuli showed the highest response, followed by IE-1 and IE-2. Figure 7 shows a comparison of the CMV-specific IFN-γ-producing CD8^+^ T-cell responses according to age (with a cut-off value of 66 years, which was the median age of the study population), gender, and diabetes status. ELISPOT assays for the 10 different CMV antigens revealed a consistent response pattern irrespective of age, gender, and diabetes.

## Discussion

Our present results showed that arterial stiffness in patients with hypertension was correlated with the frequency of senescent CD8^+^ T cells, and independently associated with the CMV pp65-specific CD8^+^ T-cell response measured by ICS. Additionally, the CMV-specific CD8^+^ T-cell responses measured by ICS and ELISPOT assays showed significant correlation and good agreement with each other irrespective of CMV antigens. Finally, ELISPOT assays for 10 different CMV antigens revealed a consistent response pattern irrespective of age, gender, and diabetes.

Several techniques can be used to measure the production of specific cytokines from T cells, including ELISPOT, enzyme-linked immunosorbent assay (ELISA), and ICS [19, 20]. The ELISPOT assay is based on the principle of the ELISA, applying detection of the antigen-stimulated secretion of cytokines trapped by an immobilized antibody, with visualization by an enzyme-coupled secondary antibody. Another approach is to use ICS with flow cytometry to detect antigen-specific T-cell responses. In this method, following brief antigen exposure, cytokines are intracellularly trapped by the addition of brefeldin A or monensin to block the secretory pathways. Subsequent permeabilization allows specific anti-cytokine fluorescent antibody to pass into the cells. The differing characteristics of these methods make it important to understand the principles of each method, to identify the appropriate methods for use in specific laboratory settings. ELISA measures the total quantity of secreted cytokines, without measuring the number of cells contributing to their production. ICS can determine the number of cells producing specific cytokines, but not their secretory activity or the amount secreted. The ELISPOT assay quantifies the frequency of cytokine-secreting cells, but not the total cytokine amount secreted.

The ELISPOT assay is typically favored for its low detection threshold, enabling ELISPOT assays to be performed with relatively low cell numbers. In contrast, ICS is favored for its more sensitive detection of cells secreting low amounts of IFN-γ [20]. These two methods reportedly show acceptable agreement with regards to measurements of virus-specific T-cell responses in a sufficient number of samples [20, 21]. Here we compared the ICS and ELISPOT assays for measuring CMV-specific T-cell responses in patients with hypertension. Our results verified that CMV-specific T-cell responses measured by ICS and ELISPOT assays showed significant correlation and good agreement with each other regardless of CMV antigens (pp65 or IE-1) in patients with hypertension.

Previous investigations of CMV-specific T-cell responses have focused on a very restricted group of CMV antigens or open reading frames (ORFs)—principally pp65 and IE-1—with the assumption that such responses are immunodominant and representative of the total T-cell response to this virus. However, experimental findings challenge this assumption. Examination of the responses to epitopes predicted by HLA-binding algorithms revealed that CD8^+^ T-cell recognition of CMV antigens is broader than anticipated, including responses to functionally and kinetically diverse antigens [22]. Our present findings expand on this previous observation, definitively demonstrating that T-cell recognition of CMV antigen is complex, often very broad, and poorly approximated by responses to any one or two CMV antigens.

Sylwester et al. examined 213 CMV ORFs and found that 151 (70%) were immunogenic for CD4^+^ T cells, CD8^+^ T cells, or both, and that 40 ORFs for CD4^+^ T cells and 33 for CD8^+^ T cells were recognized by at least 12% of subjects [23]. They further reported enormous total CMV-specific T-cell responses in seropositive subjects, comprising on average ~ 10% of both the CD4^+^ and CD8^+^ memory compartments in peripheral blood. In our present study, we used ELISPOT assays with 10 different CMV antigens to investigate CMV-specific CD8^+^ T-cell responses. We detected a clear immunogenic response, even with the lowest response to pp71, and the overall ELISPOT assay results showed a consistent response pattern irrespective of age, gender, and diabetes. We did not detect significant correlations between arterial stiffness and each antigen-specific T-cell response, probably due to the limited number of patients.

The present study has several potential limitations. First, due to limited PBMC availability, we were only able to analyze CMV-specific T-cell responses using the ELISPOT assay in a small population. Second, the immunologic analyses were conducted only in well-controlled treated hypertensive patients; thus, our present results cannot be generalized to a non-hypertensive population or to high-risk hypertensive patients. There are possibilities that normotensive subjects might show low CMV-specific T-cell responses without significant association with arterial stiffness. Third, due to the small number subjects, multivariate analysis included a limited number of essential variables including age, gender, DM, SBP, BMI, creatinine, HDL cholesterol, and hsCRP for the analysis of the relationship between CMV-specific T-cell response and arterial stiffness. Finally, our present study was not designed to examine the underlying mechanisms for the association between CMV-specific T-cell responses and arterial stiffness. Further detailed research is needed to investigate this subject.

## Conclusion

Our present results demonstrate that, in patients with hypertension, arterial stiffness was positively correlated with the frequencies of CD57^+^ and CD28^null^ cells within the total CD8^+^ T-cell population, and was independently associated with the CMV pp65-specific CD8^+^ T-cell response measured by ICS. Additionally, the CMV-specific CD8^+^ T-cell responses measured by ICS and ELISPOT assays showed significant correlation and good agreement with each other irrespective of CMV antigens. ELISPOT assays for 10 different CMV antigens revealed a consistent response pattern irrespective of age, gender, and diabetes. Overall, these results are important for guiding choices regarding the broad clinical application of CMV-specific T-cell response assays in patients with hypertension.
